# Continuous Apical Negative-Pressure Ultrasonic Irrigation (CANUI): A new concept for activating irrigants

**DOI:** 10.4317/jced.53836

**Published:** 2017-06-01

**Authors:** Pablo Castelo-Baz, Purificación Varela-Patiño, Manuel Ruíz-Piñón, Francesc Abella, Ramón Miguéns-Vila, Benjamín Martín-Biedma

**Affiliations:** 1 PhD, DDS, University of Santiago de Compostela, Facultad de Odontología, Entrerríos Street, no number. 15702, Santiago de Compostela; 2 PhD, University of Santiago de Compostela, Facultad de Odontología, Entrerríos Street, no number. 15702, Santiago de Compostela; 3 PhD. Master of Endodontics. UIC. Barcelona.; 4 DDS, University of Santiago de Compostela, Facultad de Odontología, Entrerríos Street, no number. 15702, Santiago de Compostela

## Abstract

Background: Irrigation of the root canal system is an essential step in the endodontic treatment. The aim of this article is to introduce continuous apical negative-pressure ultrasonic irrigation (CANUI), a new irrigation concept, and compare the characteristics of this new technique with current devices for activating the irrigant. Material and Methods: CANUI is designed for cleaning and disinfecting the root canal system. The device consists of a tube inside another tube that allows the continuous ultrasonic exchange of fresh irrigant, as the irrigant is simultaneously aspirated apically. The coronal and apical tubes are 0.75 and 0.3 mm in diameter, respectively. It is composed of a nickel-titanium microcannula suitable for the working length of curved canals. Results: The CANUI technique has the advantages of apical negative pressure (to avoid apical extrusion of the irrigant) and continuous ultrasonic irrigation (continuous refreshment of the irrigant and forced introduction into the canal ramifications). Conclusions: The CANUI technique could improve the irrigation technique of dentists to reach more disinfection in endodontic treatments.

** Key words:**Apical negative pressure, continuous ultrasonic irrigation, irrigation in curved canals, irrigation systems.

## Introduction

Root canal infections are typically polymicrobial and involve strong bacterial interactions (1-3). The main goal of endodontic treatment is to eliminate the infected tissue, bacteria and to fill the complex anatomy of the root canal system, in order to allow the healing of a periapical lesion or to prevent the infection of periradicular tissues. Hence, irrigation of the root canal system with antibacterial solutions is an essential step in this process (4).

The complex nature of the root canal system, with the isthmus, anastomosis, and lateral root canals, makes cleaning all areas of the root canal very complex as the anatomy prevents the irrigant from dissolving organic tissues and destroying biofilms (5). Although irrigation with a syringe and needle is still the method used most commonly (6), it fails to guarantee optimal cleaning of the root canal system (7-10).

The use of ultrasonic systems is a possible solution for cleaning root canal systems and improving disinfection; its use after mechanical preparation has been shown to reduce the number of bacteria (11). Van der Sluis *et al.* described improved cleanliness after ultrasonic passive irrigation, using a free file oscillating at ultrasonic frequencies in a canal full of sodium hypochlorite (NaOCl) (12). Gutarts *et al.* proposed the use of an ultrasonically activated needle placed inside the root canal through which NaOCl could flow, enabling continuous replenishment (5). *In vivo* studies show a high cleaning efficiency in areas that are inaccessible via instrumentation (13,14). Yet, this method can transport the irrigant farther than the distance at which the instrument acts, jeopardizing the safety of the procedure with the extrusion of NaOCl into the periapical tissues (15).

On the other hand, various studies have shown the absolute safety of negative-pressure cleaning systems compared to irrigation with a syringe or ultrasonic irrigation (16,17). In the irrigation of curved canals, ultrasonic irrigation might cause preparation irregularities (18), while the negative pressure enables the irrigants to reach the apical region, preventing the development of those irregularities.

For the above reasons, we need a device that can combine the characteristics of the negative-pressure cleaning systems and the ultrasonic irrigation systems. Therefore, the aim of this study is to introduce a device that can activate the irrigant ultrasonically in order to penetrate the canal ramifications effectively; with negative apical pressure to avoid apical extrusion of the irrigant and a nickel-titanium microcannula to transport the irrigant to the working length in curved root canals easily.

## Material and Methods

-CANUI

•Design

**Figure 1:**
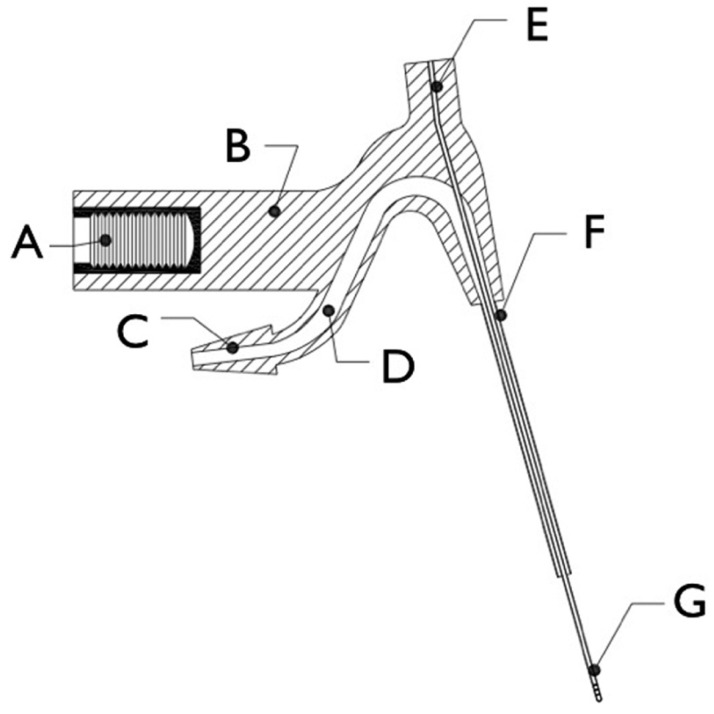
Schematic of the device: (A) female iron threaded connection; (B) plastic body; (C) plastic truncated rhomboidal joint connection; (D) cylindrical hollow conduit; (E) plastic circular joint connection; (F) stainless steel coronal cannula; and (G) apically bored nickel-titanium microcannula.

Continuous apical negative-pressure ultrasonic irrigation (CANUI) makes use of a new device for activating the irrigant in a root canal system with an ultrasonic dental unit. It consists of one tube inside another so that the same device can eject and aspirate the irrigant. The device has seven main parts (Fig. 1):

A) A threaded female adaptor attached to the body of the device that can connect to a dental ultrasonic unit;

B) A body made of hard plastic that can transmit ultrasonic vibrations and hold all the elements together;

C) A truncated rhomboidal joint that can be joined to the plastic hose that carries the irrigant, preventing liquid loss;

D) A hollow cylindrical conduit that is 1 mm in diameter overall, decreasing to 0.75 mm over the last 3 mm to increase the flow pressure of the irrigant;

E) A 0.3-mm-diameter cylindrical connection that can be connected to a dental suction system;

F) A 0.75-mm-diameter coronal cannula joined to the body of the device; and

G) A flexible nickel-titanium microcannula that allows easy access to the same working length of the curved canal. It is 0.3 mm in diameter and has six 0.05-mm holes positioned vertically in two groups of three holes each, in the apical 3 mm of the microcannula. The microcannula protrudes 8 mm from the coronal cannula (F).

•Mode of operation

The device is mounted on an ultrasonic unit, like Suprasson P5 Booster (Satelec, Acteón Group), with the power set to level 6 (equivalent to an approximate frequency of 25 kHz). A 10-ml syringe containing the irrigant is attached to the truncated rhomboidal joint (C in figure 1) with a tube. When the irrigant flow to the canal of the ultrasonic unit is activated at an intermediate power setting, it maintains a continuous irrigation flow of 3 ml/min. The microcannula is connected to the aspiration part of the dental unit to aspirate the irrigant.

**Figure 2:**
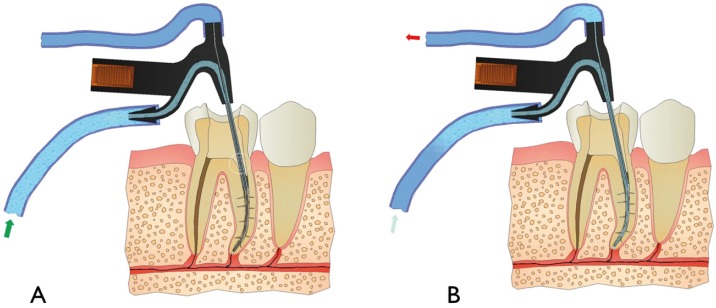
Illustration of the mode of operation showing A) the initial flow of irrigant through the device, and B) simultaneous flow and aspiration of the irrigant.

During instrumentation, the CANUI device is inserted into the coronal and middle parts of the canal to clean and disinfect the root canal system. After the instrumentation is completed, the CANUI is inserted until the apical end of the microcannula reaches a point 0.5 mm less than the working length. The inactive device is placed in the canal, and then delivery of the solution is started (Fig. 2). At this time, the device can reach its full cleaning potential.

## Discussion

-Continuous ultrasonic irrigation (CUI) to clean and disinfect the root canal system

Continuous ultrasonic irrigation (18) is a means of activating the irrigant and consists of a system that allows simultaneous continuous irrigant delivery and continuous ultrasonic activation (CUI). This CUI method is based primarily on the activation of an ultrasonically energized needle connected directly to the ultrasonic unit. The main advantages of this technique are the continuous replenishment of the solution and the ability to achieve activation without direct contact between the ultrasonic file and solution (6,18). In other words, the irrigant is delivered from the needle in an activated state, avoiding the need to insert the needle into the apical third of the root canal. The agitation of the NaOCl enhances tissue dissolution (19), and its continuous replenishment provides an uninterrupted supply of nascent chlorine for organic tissue dissolution (20). However, creating an irregularly shaped canal preparation is possible, especially in curved canals. Furthermore, if this technique is not used carefully, it can result in the undesirable extrusion of NaOCl (16).

-Apical negative pressure (ANP) to avoid extrusion of the irrigant

NaOCl combined with ethylenediaminetetraacetic acid is able to achieve the goal of chemical debridement (21,22). NaOCl can be extruded into the periapical tissues, causing inflammation, ecchymosis, hematoma, and sometimes even necrosis and paresthesia (23-25). Accordingly, any root canal irrigation delivery system that reduces the risk of NaOCl extrusion into the periapical tissues would greatly benefit patient care (16).

Desai and Himel (16), Brown *et al.* (26), Myers and Montgomery (27), and Roy and Laurence (28) reported that positive-pressure irrigation resulted in periapical extrusion, while Desai and Himel (16), Fukumoto *et al.* (17) and Mitchell (29) demonstrated that negative-pressure irrigation reduces the periapical extrusion of the irrigant. Desai and Himel (16) studied the EndoActivator, ul-trasonic needle irrigation (continuous ultrasonic irrigation), and RinsEndo. The volume extruded by the EndoActivator was very small; for continuous ultrasonic irrigation and RinsEndo, apical extrusion of the irrigant was significantly higher. Therefore, apical negative-pressure (ANP) irrigation is interesting in terms of safety.

Apical negative-pressure irrigation efficiently irrigated the root canal system up to the working length (30). This can be explained by the design of the microcannula, which eliminates vapor lock, allowing the apical exchange of irrigants. However, ANP resulted in limited activation of the irrigant in non-instrumented areas, represented by the lateral canals. Passive (PUI) and continuous (CUI) ultrasonic irrigation resulted in significantly more penetration of the irrigant into lateral canals (18,30).

-Irrigation of curved root canals

Root canals are often curved (31), making them more difficult to clean and disinfect because of the contact points of the instru-ment with the canal walls; it is necessary to bend the instrument to follow the canal curvature, which can compromise the irriga-tion efficiency and confer a risk of canal transportation (32). Rödig *et al.* (33) and Amato *et al.* (34) reported that this reduced the cleaning efficacy of several irrigation techniques. Al-Jadaa *et al.* (35), Malki *et al.* (36), and Macedo *et al.* (37) reported on the efficacy of PUI in curved canals. CUI also reaches working length in curved canals because this technique involves sufficient force to overcome the vapor lock. As a result, CUI increases the penetration of the irrigant into lateral canals and the apical third in curved roots (38). In this new concept of irrigation, CANUI brings the irrigant to the apex and the microcannula aspirates it, avoiding apical extrusion; in curved canals this is possible because the microcannula is composed of nickel-titanium.

-Clinical use

The new device must be used for irrigation after first instrumenting the entire root canal system (it is necessary instrument to at least 30.06). With our new technique to activate the irrigant, the entire root canal system can be cleaned totally and disinfected rapidly.

## Conclusions

Continuous apical negative-pressure ultrasonic irrigation represents a new approach in endodontic devices for activating the irrigant. The new design and mode of operation have the following advantages:

1. The design that allows the continuous exchange of ultrasonically activated irrigant with constant refreshment into the root canal system throughout the procedure.

2. It can clean and disinfect irregularities of the root canal system effectively with the force of continuous ultrasonic irrigation.

3. The Ni-Ti microcannula allows effective activation of the irrigant, even in curved canals.

4. Apical negative pressure avoids the risk of extrusion of the irrigant.
